# Potential Application of Bacteriophages in Enrichment Culture for Improved Prenatal *Streptococcus agalactiae* Screening

**DOI:** 10.3390/v10100552

**Published:** 2018-10-10

**Authors:** Jumpei Uchiyama, Hidehito Matsui, Hironobu Murakami, Shin-ichiro Kato, Naoki Watanabe, Tadahiro Nasukawa, Keijiro Mizukami, Masaya Ogata, Masahiro Sakaguchi, Shigenobu Matsuzaki, Hideaki Hanaki

**Affiliations:** 1 School of Veterinary Medicine, Azabu University, Kanagawa 252-5201, Japan; h-murakami@azabu-u.ac.jp (H.M.); n.watanabe1011@gmail.com (N.W.); dv1804@azabu-u.ac.jp (T.N.); mizukami@azabu-u.ac.jp (K.M.); a15121@azabu-u.ac.jp (M.O.); sakagum@azabu-u.ac.jp (M.S.); 2 Kitasato Institute for Life Sciences, Kitasato University, Tokyo 108-8641, Japan; m_hidehito@yahoo.co.jp (H.M.); hanakihideaki@yahoo.co.jp (H.H.); 3 Research Institute of Molecular Genetics, Kochi University, Kochi 783-8502, Japan; katoshin@kochi-u.ac.jp; 4 Kochi Medical School, Kochi University, Kochi 783-8505, Japan; matuzaki@kochi-u.ac.jp

**Keywords:** phage, *Enterococcus faecalis*, *Streptococcus agalactiae*, culture enrichment

## Abstract

Vertical transmission of *Streptococcus agalactiae* can cause neonatal infections. A culture test in the late stage of pregnancy is used to screen for the presence of maternal *S. agalactiae* for intrapartum antibiotic prophylaxis. For the test, a vaginal–rectal sample is recommended to be enriched, followed by bacterial identification. In some cases, *Enterococcus faecalis* overgrows in the enrichment culture. Consequently, the identification test yields false-negative results. Bacteriophages (phages) can be used as antimicrobial materials. Here, we explored the feasibility of using phages to minimize false-negative results in an experimental setting. Phage mixture was prepared using three phages that specifically infect *E. faecalis*: phiEF24C, phiEF17H, and phiM1EF22. The mixture inhibited the growth of 86.7% (26/30) of vaginal *E. faecalis* strains. The simple coculture of *E. faecalis* and *S. agalactiae* was used as an experimental enrichment model. Phage mixture treatment led to suppression of *E. faecalis* growth and facilitation of *S. agalactiae* growth. In addition, testing several sets of *S. agalactiae* and *E. faecalis* strains, the treatment with phage mixture in the enrichment improved *S. agalactiae* detection on chromogenic agar. Our results suggest that the phage mixture can be usefully employed in the *S. agalactiae* culture test to increase test accuracy.

## 1. Introduction

*Streptococcus agalactiae* (also called group B *Streptococcus*) is vertically transmitted to the newborn during delivery, and can cause neonatal infections [1,2]. Common early-onset diseases caused by this organism in infants include sepsis and pneumonia, and (rarely) meningitis [1,2]. To prevent such infections, a prenatal *S. agalactiae* culture test is recommended in the late stage of pregnancy [1,2]. In the case of a positive test result, the pregnant carrier is prophylactically treated with antibiotics to prevent vertical transmission of *S. agalactiae* during the intrapartum period [1,2].

For the *S. agalactiae* culture test, the Centers for Disease Control and Prevention highly recommend an enrichment culture, followed by conventional *S. agalactiae* identification [3,4]. In the culture test, a swab is taken from the vaginal and anorectal areas, and the samples are inoculated and cultured in an enrichment culture broth selective for *S. agalactiae*. After the enrichment culture, bacterial identification is performed, e.g., using the Christie–Atkins–Munch-Petersen test, serologic identification, growth on chromogenic agar, and nucleic acid amplification [4]. However, although a selective culture broth is used for the enrichment culture, *S. agalactiae* is poorly recovered along with overgrowth of *Enterococcus faecalis* in some cases [5,6,7,8]. This may lead to false-negative results in the subsequent identification tests [5,6,7,8]. To address this problem, selective antimicrobial agents to be included in the enrichment broth should be reevaluated.

Bacteriophages (phages), i.e., bacterial viruses, infect specific bacteria. Some phages infect and lyse bacteria at the specificity level of species and strains. These phage characteristics were used to eliminate most cells in a bacterial population and facilitate the isolation of less prevalent environmental bacteria that produce novel bioactive compounds [9]. Phage applicability for the isolation of food-poisoning microbes in the food microbiology field was also examined [10]. Hence, potentially, phage application might also be used to reduce the unwanted growth of *E. faecalis* in an *S. agalactiae* enrichment culture and to facilitate *S. agalactiae* detection in clinical microbiology. Indeed, phages that specifically infect *E. faecalis* were isolated from environmental samples, such as sewage and canal water [11,12,13]. In the current study, we examined the applicability of *E. faecalis*-specific phages to suppress *E. faecalis* growth in an *S. agalactiae* enrichment culture in an experimental setting.

## 2. Materials and Methods

### 2.1. Bacteria, Phages, and Culture Media

Strains of *E. faecalis* (*n* = 30), *S. agalactiae* (*n* = 7), *Enterococcus avium* (*n* = 5), and *Enterococcus faecium* (*n* = 5) were isolated from vaginal swabs using the Chrom-ID Strepto B test (bioMérieux, Marcy-l’Étoile, France). The swabs were obtained after random sampling at local hospitals in eastern Japan (Table S1). Bacteria were cultured at 37 °C under aerobic or microaerobic (i.e., 5% CO_2_) conditions, as appropriate, based on their specific growth requirements (Table S1).

Phage phiEF24C was isolated and characterized, as described elsewhere [12,14,15]. Phage phiEF17H was newly isolated from canal water in Kochi (Japan). Phage phiM1EF22 was newly isolated from sewage water in Tokyo (Japan) (Table S2). The isolation procedures are described in Reference [12]. *E. faecalis* strains KUEF01, KUEF25, and KUEF27, described in Table S1, were used as host bacteria for phages phiEF24C, phiEF17H, and phiM1EF22, respectively, for phage amplification and plaque assay. Bacterial-phage suspensions were cultured aerobically at 37 °C.

*Enterococcus* spp. and phages were cultured in tryptic soy broth or agar (TSA), and *S. agalactiae* was cultured in Todd–Hewitt broth (THB), unless stated otherwise. Granada-type broth (GtB; 25.0 g/L proteose peptone no. 3, 14.0 g/L soluble starch, 2.5 g/L glucose, 1.0 g/L pyruvic acid sodium salt, 0.1 g/L cysteine hydrochloride, 0.3 g/L magnesium sulfate, 11.0 g/L 3-(*N*-morpholino)propane sulfonic acid, 10.7 g/L disodium hydrogen phosphate, 0.5 mg/L crystal violet, 10 mg/L colistin sulfate, 10 mg/L metronidazole, and 15 mg/L nalidixic acid, pH 7.4) was originally prepared as the *S. agalactiae* enrichment broth [16,17]. Alternatively, the pigmented enrichment Lim broth (modified Lim broth; Kyokuto Pharmaceutical Industrial, Tokyo, Japan) was used as an *S. agalactiae* enrichment broth. Unless stated otherwise, all culture media were purchased from Becton, Dickinson, and Co. (Franklin Lakes, NJ, USA). All chemicals and reagents were purchased from Nacalai Tesque (Kyoto, Japan) and FUJIFILM Wako Pure Chemical (Osaka, Japan).

### 2.2. Phage Genome Sequencing and Analysis

After phage amplification, phage particles were purified from 500 mL of phage lysate by CsCl density-gradient centrifugation, as described elsewhere [18]. Phage genomic DNA was then prepared by phenol–chloroform extraction of the collected purified phage band [18]. A shotgun library was prepared for each phage DNA using the GS FLX Titanium rapid library preparation kit (Roche Diagnostics, Indianapolis, IN, USA), according to the manufacturer’s instructions. The libraries were analyzed using a GS Junior 454 sequencer (Roche Diagnostics, Risch-Rotkreuz, Switzerland). The sequence reads were assembled using the 454 Newbler software (version 3.0; 454 Life Sciences, Branford, CT, USA). The genomes were annotated using a prokaryotic genome annotation pipeline, DFAST (https://dfast.nig.ac.jp/) [19,20]. The phiEF17H and phiM1EF22 genome sequences were deposited in GenBank under the accession numbers AP018714 and AP018715, respectively.

The genome sequences were analyzed using nucleotide Basic Local Alignment Search Tool (BLASTn) at the National Center for Biotechnology Information (NCBI; https://blast.ncbi.nlm.nih.gov/Blast.cgi?PROGRAM=blastn&PAGE_TYPE=BlastSearch&LINK_LOC=blasthome; last accessed: 5 May 2018). Moreover, the genomes of phages belonging to the family *Myoviridae* subfamily *Spounavirinae* were downloaded from GenBank (last accessed: 20 September 2018), and the viral phylogeny was analyzed using a proteomic tree analysis tool, ViPTree version 1.0 [21].

### 2.3. Multilocus Sequence Typing (MLST) of *E. faecalis* Strains

*E. faecalis* strains were cultured overnight, bacterial DNA was extracted, and MLST analysis was performed, according to the procedures described elsewhere [22]. The sequence alleles were analyzed using the *E. faecalis* MLST database (https://pubmlst.org/efaecalis/; last accessed: 5 January 2018) to designate sequence types (STs) [23]. The concatenating allele sequences were analyzed using MEGA 7.0.18, and sequence alignment implemented in ClustalW was followed by phylogenetic tree construction using the unweighted pair group with arithmetic mean (UPGMA) method [24].

### 2.4. Examination of Antibacterial Activity of *E. faecalis* to *S. agalactiae*

The anti-*S. agalactiae* activity of *E. faecalis* was examined using a spot-on-lawn assay, as described elsewhere [25]. Briefly, 200 μL of overnight bacterial culture of a single *S. agalactiae* strain was mixed with a melted 0.5% (*w*/*v*) soft agar and plated onto 1.5% (*w*/*v*) agar. One microliter of *E. faecalis* overnight culture was spotted on the solidified top agar. After incubation overnight at 37 °C in a microaerophilic condition, *S. agalactiae* growth around the spotted *E. faecalis* was examined.

### 2.5. Analysis of Phage Lytic Activity

The phage host range was determined by a streak test, as described elsewhere [12,15]. Briefly, 200 μL of overnight bacterial culture of a single bacterial strain was mixed with a melted 0.5% (*w*/*v*) soft agar and plated onto 1.5% (*w*/*v*) agar. The phage suspension (ca. 1.0 × 10^8–9^ plaque-forming units (PFU)/mL) was streaked onto the solidified top agar. After incubation overnight at 37 °C, bacterial lysis, with or without plaque formation, was examined.

### 2.6. Analysis of Bacterial Densities in *S. agalactiae* and *E. faecalis* Coculture with Phage Mixtures

A rifampicin-resistant mutant clone of *S. agalactiae* was isolated by aerobically culturing *S. agalactiae* strain KUGBS2 on TSA containing 20 μg/mL rifampicin at 37 °C for two days. The putative mutant clones were repurified at least three times; each repurification round was repeated for one day under the same incubation conditions. One resultant rifampicin-resistant mutant clone of strain KUGBS2 was obtained and was tentatively designated as strain KUGBS2rif. *S. agalactiae* strain KUGBS2rif and *E. faecalis* strain KUEF08 were cultured individually until an optical density of 0.4–0.6 at 600 nm was attained. After diluting with the enrichment broth, suspensions of 3.0 × 10^4^ colony-forming units (CFU)/mL *S. agalactiae* strain KUGBS2rif and 3.0 × 10^7^ CFU/mL *E. faecalis* strain KUEF08 were prepared. Each phage suspension was diluted with THB to ca. 3.0 × 10^6^ PFU/mL or 3.0 × 10^4^ PFU/mL. By mixing equal volumes of phage suspensions at the same dilution, mixtures of two different dilutions of phages were prepared.

For the experiment, 100 μL each of *S. agalactiae* strain KUGBS2rif and *E. faecalis* strain KUEF08, and 300 μL of phage mixture were added to 10 mL of the enrichment broth. As negative controls, the same volume of THB was added instead of bacterial suspensions and/or phage suspensions. The mixtures were microaerobically incubated at 37 °C for 24 h. Total bacterial density and *S. agalactiae* strain KUGBS2rif and *E. faecalis* strain KUEF08 densities were determined. Total bacterial densities were determined on TSA. TSA supplemented with 20 μg/mL rifampicin and *Enterococcus*-selective agar (EF agar base “Nissui”; Nissui Pharmaceutical Co., Tokyo, Japan) were used to determine the densities of *S. agalactiae* strain KUGBS2rif and *E. faecalis* strain KUEF08, respectively. *S. agalactiae* strain KUGBS2rif did not grow on the *Enterococcus*-selective agar; *E. faecalis* strain KUEF08 did not grow on TSA containing 20 μg/mL rifampicin.

### 2.7. Detection of Bacteria on Chromogenic Selective Agar after *S. agalactiae* and *E. faecalis* Coculture with Phage Mixtures

*S. agalactiae* and *E. faecalis* were cultured individually until an optical density of 0.4–0.6 at 600 nm was obtained. *S. agalactiae* and *E. faecalis* cultures were diluted with THB to ca. 3.0–5.0 × 10^4^ CFU/mL and ca. 3.0 × 10^7^ CFU/mL, respectively. After dilution of individual phage suspensions in THB to ca. 1.0 × 10^7^ PFU/mL, the phage mixture was prepared by mixing equal volumes of the diluted phage suspensions.

For the experiment, 30 μL each of bacterial suspensions of *S. agalactiae* and *E. faecalis*, and 30 μL of phage mixture were added to 3 mL of the enrichment broth. As a negative control, the same volume of THB was added instead of the phage mixture. After 24-h incubation at 37 °C, a loopful of the suspension was inoculated on the Chrom-ID Strepto B agar (BioMérieux, Marcy-l'Étoile, France). After 24-h incubation at 37 °C in darkness, colony color and appearance on agar plates were examined. All incubations were carried out under microaerophilic conditions.

### 2.8. Statistical Analysis

The data were statistically analyzed using EZR (Saitama Medical Center, Jichi Medical University, Saitama, Japan), which is a graphical user interface for R (The R Foundation for Statistical Computing, Vienna, Austria) [26]. Student’s *t*-tests were used to analyze differences between bacterial densities in different treatments. A *p*-value < 0.01 was considered to indicate a statistically significant difference.

## 3. Results and Discussion

### 3.1. Phage Characteristics

**Figure 1:**
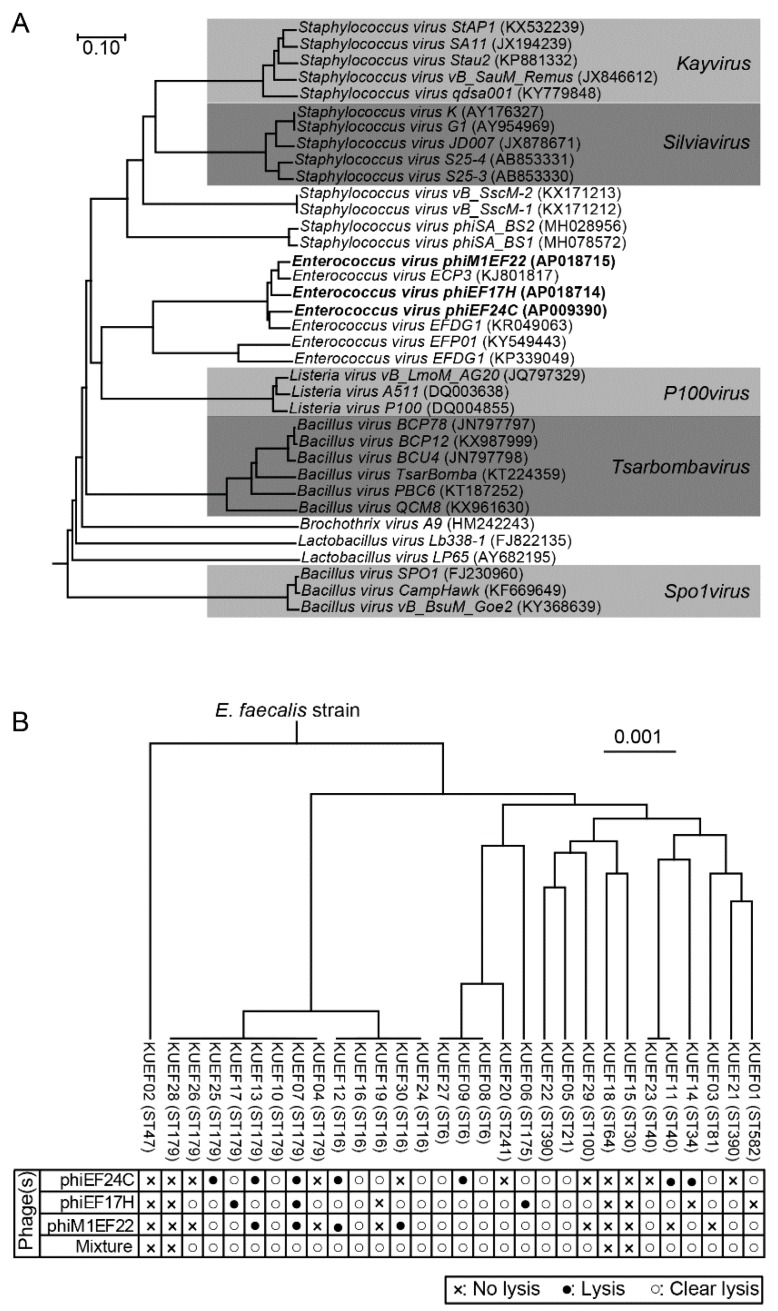
*Enterococcus* phages phiEF24C, phiEF17H, and phiM1EF22 and their lytic activity to various *E. faecalis* strains. (**A**) Viral proteomic trees of *Enterococcus* phages phiEF24C, phiEF17H, and phiM1EF22 in the family *Myoviridae* subfamily *Spounavirinae*. *Enterococcus* phages phiEF24C, phiEF17H, and phiM1EF22 are shown in bold. Phage taxonomical names are shown followed by the GenBank accession number in parentheses. The phages belonging to a certain viral genus are shown in grey highlight, on which the viral genus names are indicated. (**B**) *E. faecalis* strains isolated from vaginal swabs and their sensitivity to phages. Phylogenetic tree of *E. faecalis* strains was constructed based on the concatenated multilocus sequence typing (MLST) alleles. In the phylogenetic tree, *E. faecalis* strain names are followed by sequence types (STs) in brackets. Phage sensitivities to each phage and phage mixture are shown below the phylogenetic tree.

Phages phiEF24C, phiEF17H, and phiM1EF22 were used in the current study. Phage phiEF24C, one of the best-studied *Enterococcus* phages, is classified into the family *Myoviridae* subfamily *Spounavirinae* [12,14,15]. The other two phages, phiEF17H and phiM1EF22, were newly isolated and their whole genomes were sequenced. The whole-genome sequence similarity analysis using the BLASTn showed that phages phiEF17H and phiM1EF22 are similar to phage phiEF24C (Table S3). Thus, they were considered to be classified into the family *Myoviridae* subfamily *Spounavirinae* [27]. Moreover, the phylogenetic relationship of these three phages used in this study was analyzed with 33 other phages of the family *Myoviridae* subfamily *Spounavirinae*. Phages sharing this particular viral taxonomy of the family *Myoviridae* subfamily *Spounavirinae* are highly virulent toward host bacteria [28]. The tree showed that phages phiEF24C, phiEF17H, and phiM1EF22 were phylogenetically clustered but slightly different from each other among the *Enterococcus* phages belonging to this viral subfamily (Figure 1A). These *Enterococcus* phages may be categorized into a new virus genus in this subfamily.

### 3.2. Characteristics of *E. faecalis* Strains Isolated from Vaginal Swabs

The genetic background of *E. faecalis* strains isolated from the vaginal swabs in this study was examined. MLST analysis of the *E. faecalis* vaginal swab isolates revealed that 43.3% (13/30) of *E. faecalis* strains were phylogenetically closely related, representing either ST16 or ST179 (Figure 1B). The remaining strains (56.7% (17/30)) were genetically diverse. Moreover, *E. faecalis* strains do not seem to show antibacterial activity (e.g., bacteriocin production) to *S. agalactiae*, while some of them are able to show antibacterial activity to a variety of bacteria [5,29]. Testing the antibacterial activity to *S. agalactiae* strains using the spot-on-lawn assay, no anti-*S. agalactiae* activity was observed among these *E. faecalis* strains.

### 3.3. Phage Lytic Spectrum and Phage Mixture

Phage lytic activity was examined with a streak test using these *E. faecalis* strains (Figure 1B). Phages phiEF24C, phiEF17H, and phiM1EF22 showed lytic activity toward 63.3% (19/30), 76.7% (23/30), and 66.7% (20/30), respectively, of the tested *E. faecalis* strains. Because these phages have slightly different genomes, they have different host ranges to *E. faecalis* strains. Moreover, lytic activities of the three phages with other bacterial vaginal swab isolates (*E. avium*, *E. faecium*, and *S. agalactiae* strains) were also examined, but no lytic activity was observed.

Phages phiEF24C, phiEF17H, and phiM1EF22 lysed different *E. faecalis* strains and also some common strains. Theoretically, a combination of these three phages lysed a broader range of *E. faecalis* strains than any single phage tested. A phage mixture containing the three phages was prepared by mixing phage particles in a 1:1:1 ratio, which was also used in the following experiments. The lytic spectrum of the phage mixture was then examined using the streak test described above. The phage mixture showed lytic activity toward 86.7% (26/30) of *E. faecalis* strains tested (Figure 1B). Four *E. faecalis* strains were not lysed by the phages, namely, KUEF02 (MLST ST47), KUEF18 (ST64), KUEF15 (ST30), and KUEF28 (ST179). Because these bacterial strains may have several phage-resistant mechanisms [30], they cannot be lysed with these phages in the phage mixture. In addition, the phage mixture did not show any lytic activity with the other tested bacteria, i.e., *E. avium*, *E. faecium*, and *S. agalactiae*, as seen in the assessments of lytic activity of individual phages as above.

The phage mixture may have been contaminated with anti-*S. agalactiae* agents during phage mixture preparation (i.e., during phage propagation on the *E. faecalis* host). The antimicrobial activity of the phage mixture to *S. agalactiae* was examined. Incubation of the phage mixture with *S. agalactiae* for 30 min and 24 h did not influence bacterial viability, compared with a THB-treated negative control (Figure S1), excluding the possibility of phage mixture contamination with anti-*S. agalactiae* substances. Thus, the phage mixture could be used as anti-*E. faecalis* agents in the enrichment broth to examine the suppression of *E. faecalis* growth in *S. agalactiae* enrichment culture.

### 3.4. Effect of Phage Mixture on *S. agalactiae* and *E. faecalis* Cell Densities in Experimental Enrichment Cultures

The vaginal microbiome of pregnant women is majorly composed of *Lactobacillus* spp., together with the bacteria of phyla *Actinobacteria*, *Firmicutes*, and others in minor quantities [31,32,33]. During the culture of swab seed in *S. agalactiae*-selective enrichment broth, the growth of the majority of vaginal microflora is inhibited and a minority, including *S. agalactiae*, *E. faecalis*, and other bacteria, are selectively grown when they are presented in the swab. In this study, a situation in which *E. faecalis* thrives and *S. agalactiae* grows poorly was mimicked in an experimental setting, using a simple coculture of *E. faecalis* and *S. agalactiae* in *S. agalactiae*-selective enrichment broth. Using the model, the effects of the phage mixture were then examined.

The simple coculture of *E. faecalis* and *S. agalactiae* in *S. agalactiae*-selective enrichment broth was used as an experimental enrichment model, which mimicked the situation of poor recovery of *S. agalactiae*. In the experimental enrichment model, *S. agalactiae* and *E. faecalis* cell densities were monitored in the presence and absence of the phage mixture over time. To mimic the situation in which *S. agalactiae* was poorly recovered, *S. agalactiae* strain KUGBS2rif and *E. faecalis* strain KUEF08 were inoculated into GtB at 3.0 × 10^2^ CFU/mL and 3.0 × 10^5^ CFU/mL, respectively. Either of the two dilutions of phage mixture (at a multiplicity of infection (MOI) of each phage of 10^−1^ and 10^−3^ to *E. faecalis*) was added. As a negative control, THB was used instead of the phage mixture.

**Figure 2:**
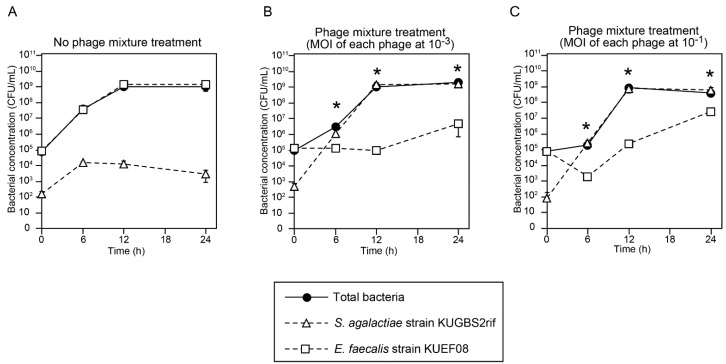
Growth of *E. faecalis* and *Streptococcus agalactiae* cocultures in the presence or absence of phage mixtures in Granada-type broth (GtB). No phage treatment (**A**); or treatment with phages at 10^−3^ (**B**) or at 10^−1^ (**C**) multiplicities of infection (MOIs) of each phage to *E. faecalis*. The means with standard deviations were calculated from triplicate experiments, and are plotted as points with error bars. Time points at which *S. agalactiae* density was significantly higher than that of *E. faecalis* are indicated by asterisks (*p* < 0.01; Student’s *t*-test).

Bacterial cell densities (total bacteria, *S. agalactiae*, and *E. faecalis*) were then monitored over time (Figure 2). Firstly, based on the determined total bacteria numbers in both the negative control and phage treatment groups, bacteria grew exponentially for up to 12 h, following which the cultures entered the stationary phase of growth (Figure 2). Hence, incubation for 12–24 h was sufficient to achieve bacterial enrichment in that particular experimental setting. Moreover, *S. agalactiae* cell densities were compared with *E. faecalis* cell densities at different time points in the negative control and phage treatment groups. In the negative control group (i.e., no phage treatment), *E. faecalis* densities were significantly higher than *S. agalactiae* at incubation for 6, 12, and 24 h (*p* < 0.01) (Figure 2A). In contrast, in the phage treatment groups at MOIs of 10^−1^ and 10^−3^ of each phage, the cell density of *E. faecalis* was significantly lower than that of *S. agalactiae* at incubation for 6, 12, and 24 h in the phage treatment groups (*p* < 0.01) (Figure 2B,C).

We also evaluated the effectiveness of phage treatment in the experimental enrichment model using the commercially available pigmented enrichment Lim broth (modified Lim broth) (Figure S2). The experiments were performed using the same method described above. Inhibition of *E. faecalis* growth, compared with the untreated group, was observed in the phage treatment groups at both MOIs of each phage tested (i.e., 1 and 10^−2^) (Figure S3). These results indicate that the phage mixture inhibited *E. faecalis* growth and facilitated *S. agalactiae* growth in both *S. agalactiae* enrichment broths.

### 3.5. Efficient Detection of *S. agalactiae* after Experimental Enrichment Culture in the Presence of the Phage Mixture

**Figure 3:**
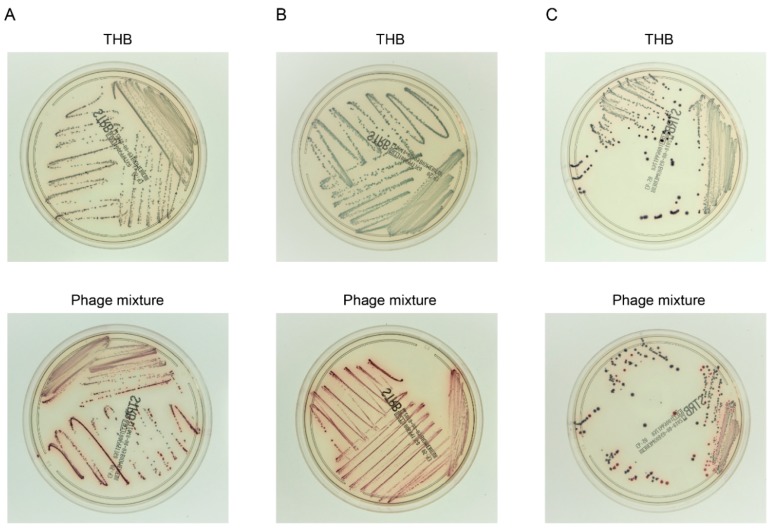
Bacterial identification on chromogenic agar after experimental enrichment coculture of *S. agalactiae* and *E. faecalis*. Combinations of single strains of *E. faecalis* and *S. agalactiae* were used to inoculate GtB, and were cultured in the presence of the phage mixture (MOI of each phage to *E. faecalis*: 10^−1^) or Todd–Hewitt broth (THB). After enrichment culture, aliquots were spread on the chromogenic agar, and the resultant bacterial colonies were evaluated. Colonies of *S. agalactiae* and *E. faecalis* are red and blue, respectively. Top and bottom panels show photographs of chromogenic agar plates inoculated with enriched cultures treated with THB or phage mixtures, respectively. Representative data for three out of five *S. agalactiae*–*E. faecalis* sets are shown, namely, KUGBS2–KUEF08 (**A**), KUGBS1–KUEF24 (**B**), and KUGBS6–KUEF26 (**C**).

In the *S. agalactiae* culture test, bacteria are generally identified in a culture aliquot after enrichment culture. Consequently, we then evaluated the efficiency of *S. agalactiae* identification after the experimental enrichment culture. As the identification assay, we used growth on the *S. agalactiae* chromogenic agar, in which *S. agalactiae* colonies are distinguished from *E. faecalis* colonies based on color. Five *S. agalactiae*–*E. faecalis* sets were tested: KUGBS2–KUEF08, KUGBS1–KUEF24, KUGBS6–KUEF26, KUGBS4–KUEF21, and KUGBS7–KUEF29. *E. faecalis* and *S. agalactiae* strains were first inoculated at a 100:1 ratio in the Granada-type enrichment broth; then, the phage mixture was added (at an MOI of each phage at 10^−1^ to *E. faecalis*), and the cultures were incubated. Enrichment culture aliquots were plated on chromogenic agar, and the resultant colony appearance was evaluated (Figure 3). After enrichment of all phage-treated *S. agalactiae*–*E. faecalis* sets, *S. agalactiae* colonies were dominant on the agar plates. By contrast, in enrichment cultures without phage treatment, only a few *S. agalactiae* colonies were observed on the chromogenic agar, while *E. faecalis* colonies were dominant. The same experiment was performed using the modified Lim broth and several *S. agalactiae*–*E. faecalis* combinations (Figure S4). The data were in agreement with observations made using the GtB. Thus, the phage mixture treatment is believed to improve the *S. agalactiae* culture test by inhibiting the undesirable growth of *E. faecalis*.

### 3.6. Phage Application Potential in the Clinical Setting

In the current study, we showed that a specific phage mixture effectively inhibited the growth of *E. faecalis* in an *S. agalactiae* culture test in the experimental setting. Before clinical application and reagent manufacture, it is necessary to discuss issues such as the phage composition in the phage mixture, the usage per assay (i.e., volume and phage density), storage of the phage mixture, and production cost.

As a first consideration, strains insensitive to the phage mixture may occur at a higher than expected rate in the clinical setting. To address this issue, phage sensitivity of *E. faecalis* strains should be constantly examined. The effective spectrum of phages in a mixture can be modified by replacing and/or adding other phages, including newly isolated phages, naturally evolved phages, and/or genetically modified phages [34,35]. For example, by adding six newly isolated phages to the phage mixture developed in the current study, we increased the inhibition efficiency among the tested *E. faecalis* strains to 96.7% (29/30). Hence, updating the composition of the phage mixture will help address the problem associated with the phage-insensitive strains.

Next, we considered the usage per assay. Assuming that the volume of the enrichment broth is ca. 3–4 mL, a drop of phage mixture suspension (i.e., 30–50 μL of a solution of ca. 1.0 × 10^7^ PFU/mL of each phage) would suffice for an assay. Such usage per assay is in line with the usage of the phage mixture in the experiments performed in the current study (Figure 3). Thus, the phage mixture complies with the usage expected in a clinical setting.

Importantly, the quality of the phage mixture should be guaranteed to allow its commercialization. Phages are generally stable at 4 °C in culture media for a certain period of time [36,37], which suggests that phage products can be distributed on a market scale using the cold chain. Accordingly, we examined the infectious density of each phage solution during storage at 4 °C for 270 days; we did not observe any substantial reduction in phage particle density during this period (Figure S5). The shelf life of culture medium is generally up to six months [38]; no loss in sensitivity in *S. agalactiae* enrichment broths was observed after at least four months [39]. Thus, the stability of the phage mixture appears to be in line with the storage of the enrichment broths.

Finally, the use of phage mixture should be cost-effective. Phage production on a bioreactor scale was recently investigated because of increased interest in phage therapy. Consequently, the cost of phage production is estimated to be $4.4 × 10^−13^/phage particle [40,41,42]. Hence, the calculated cost of the phage mixture per assay in the current study is only $2.2 × 10^−7^ ($4.4 × 10^−13^/phage particle multiplied by the number of phage particles in the mixture, i.e., 1.0 × 10^7^ PFU/mL × 50 μL). A number of commercial phage companies were established worldwide [43]. Thus, in theory, phage mixtures may be produced for the *S. agalactiae* culture test in a cost-effective manner.

After commercialization of the phage mixture, special attention should be devoted to the biological characteristics of phages to avoid laboratory accidents, because clinical tests are at high risk of interference by phage contamination via aerosols. For example, *Enterococcus* phages may contaminate clinical *E. faecalis* culture tests and interfere with the test results in a clinical laboratory. To avoid such accidents, appropriate good laboratory hygiene and sterilization practices should be instated when preparing to use phage products. Such measures can include aseptic handling at a specified bench (e.g., safety cabinet), change of gloves and ultraviolet (UV) irradiation of the bench after usage, and storage in a specific cabinet [44]. We believe that the phage mixture is appropriate for use in the clinical laboratory, as long as the knowledge about phage products is disseminated and implemented. Thus, we anticipate that the phage mixture will become commercially available for *S. agalactiae* enrichment broths in the future and will be used in the clinical setting.

## Supplementary Materials

The following are available online at http://www.mdpi.com/1999-4915/10/10/552/s1: Table S1: Bacterial strains used in the current study; Table S2: Phages used in the current study; Table S3: Genome sequences of *Enterococcus* phages used in this study; Figure S1: Examination of the anti-*S. agalactiae* effects of the phage mixture; Figure S2: Color change of the pigmented modified Lim broth with bacterial growth; Figure S3: Effects of the phage mixture on bacteria inoculated in a commercially available *S. agalactiae* enrichment broth; Figure S4: Bacterial identification on chromogenic agar after experimental enrichment coculture of *S. agalactiae* and *E. faecalis*; Figure S5: Phage stability.
