# Supplementary material for: Potential Application of Bacteriophages in Enrichment Culture for Improved Prenatal Streptococcus agalactiae Screening
Source: Viruses. 2018 Oct 10;10(10):552. doi: 10.3390/v10100552 (PMC6213948; doi:10.3390/v10100552)
Supplement: Supplementary file 1 [file viruses-10-00552-s001.pdf]

Table S1. Bacterial strains used in the current study.

Bacterial genus and species	Strain	Culture condition[1]
E. faecalis	KUEF01, KUEF02, KUEF03, KUEF04, KUEF05, KUEF06, KUEF07, KUEF08, KUEF09, KUEF10, KUEF11, KUEF12, KUEF13, KUEF14, KUEF15, KUEF16, KUEF17, KUEF18, KUEF19, KUEF20, KUEF21, KUEF22, KUEF23, KUEF24, KUEF25, KUEF26, KUEF27, KUEF28, KUEF29, KUEF30	Aerobically at 37°C
E. avium	KUEavium1, KUEavium2, KUEavium3, KUEavium4, KUEavium5	Aerobically at 37°C
E. faecium	KUEFum1, KUEFum2, KUEFum3, KUEFum4, KUEFum5	Aerobically at 37°C
S. agalactiae	KUGBS1, KUGBS2, KUGBS3, KUGBS4, KUGBS5, KUGBS6, KUGBS7	Microaerobically at 37°C, unless otherwise stated

[1], *S. agalactiae* grows under microaerophilic conditions better than under aerobic conditions.

Table S2. Phages used in the current study.

Phage	Description
phiEF24C	Phage phiEF24C has been previously isolated and extensively characterized (12, 14, 15).
phiEF17H	Phage phiEF17H was isolated from canal water in Kochi, Japan. It was isolated using a clinical *E. faecalis* strain EF17 obtained from the Kochi University Hospital (Kochi, Japan). *E. faecalis* strain EF17 has been described previously (12, 14, 15).
phiM1EF22	Phage phiM1EF22 was isolated from sewage water in Tokyo, Japan. It was isolated using *E. faecalis* strain KUEF22 isolated from the vaginal swabs at the Kitasato University Hospital (Kanagawa, Japan).

Table S3. Genome sequences of *Enterococcus* phages used in this study.

Phage[1]	No of reads	Sequence depth	Genome size (bp)	G+C content (%)	No. of CDSs	No. of tRNAs	GenBank accession No.	BLASTn analysis to *Enterococcus* phage phiEF24C genome				
								Max score	Total score	Query cover	E value	Ident
Sequenced phage genomes in this study												
**phiEF17H**	20,015	58	143,638	35.96	204	7	AP018714	39352	2.37E+05	90%	0	98%
**phiM1EF22**	15,368	50	143,046	35.95	204	5	AP018715	35345	2.43E+05	92%	0	97%
Reported phage genome sequences												
**phiEF24C**			142,072	35.74	221	5	AP009390	2.56E+05	2.91E+05	100%	0	100%
EFLK1			130,952	35.89	208	0	KR049063	38358	2.31E+05	94%	0	98%
ECP3			145,518	35.91	219	5	KJ801817	35352	2.47E+05	91%	0	97%
EFDG1			147,589	37.20	211	24	KP339049	5946	46626	51%	0	77%
EFP01			155,053	37.02	192	0	KY549443	7638	47102	48%	0	75%

[1], Phages used in this study are shown in bold.

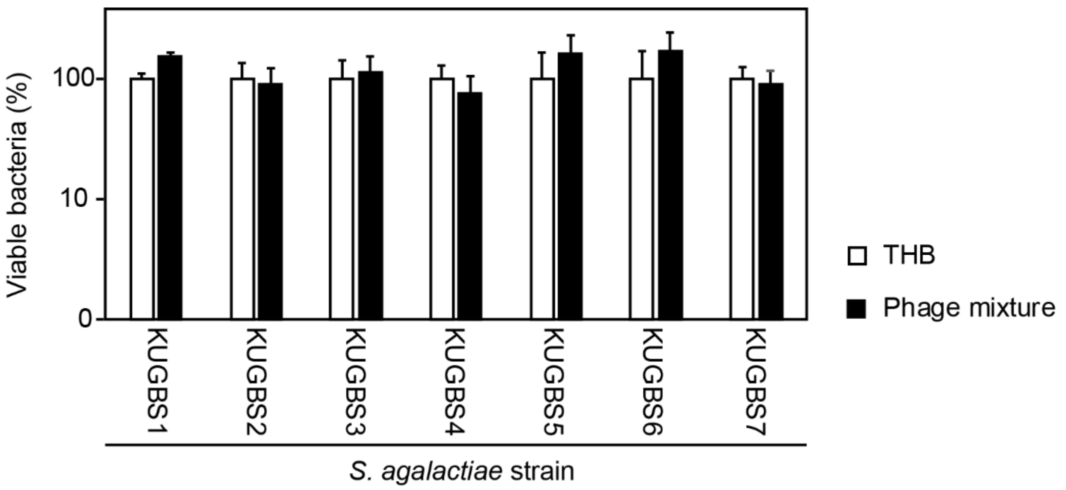

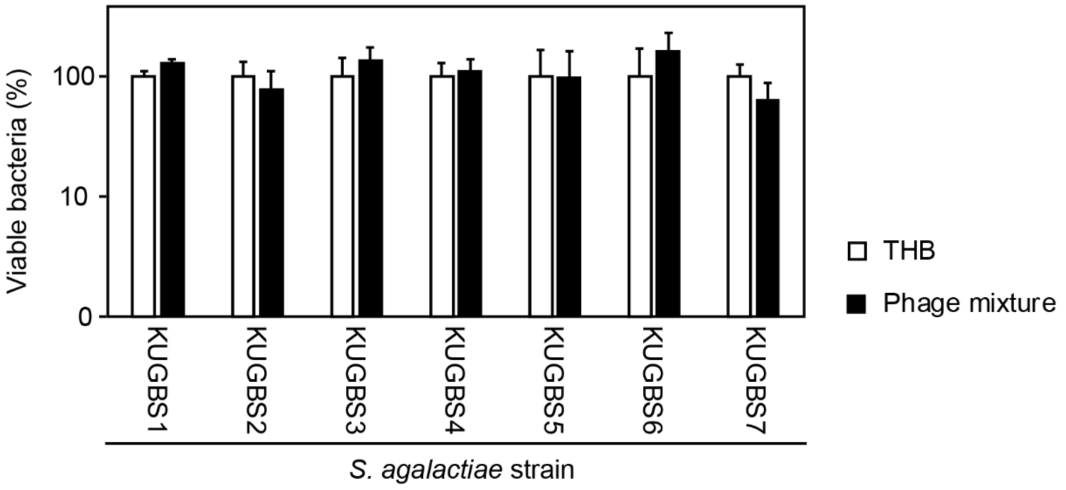

Figure S1. Examination of the anti-*S. agalactiae* effects of the phage mixture. Phage suspensions were diluted with THB to ca. 1.0×10^7 PFU/mL, and the phage mixture was prepared by mixing equal volumes of diluted phage suspension. Then, 5 μL of *S. agalactiae* suspension ($1.0–3.0 \times 10^4$ CFU/mL), which was cultured until optical density of 0.4–0.6 at 600 nm and diluted with THB, were mixed with 495 μL of the phage mixture or THB. The mixture was incubated on a stand in the microaerobic condition for 30 min (top) and 24 h (bottom) at 37°C. After incubation, bacterial density was determined by plating the suspension on THB agar. The mean bacterial density in the THB treatment group was set as 100%. The data are presented as means (bar graphs) with standard deviations (error bars). No significant differences between phage mixture-treated and THB-treated cultures of seven *S. agalactiae* strains tested were noted (*t* test, $p < 0.01$).

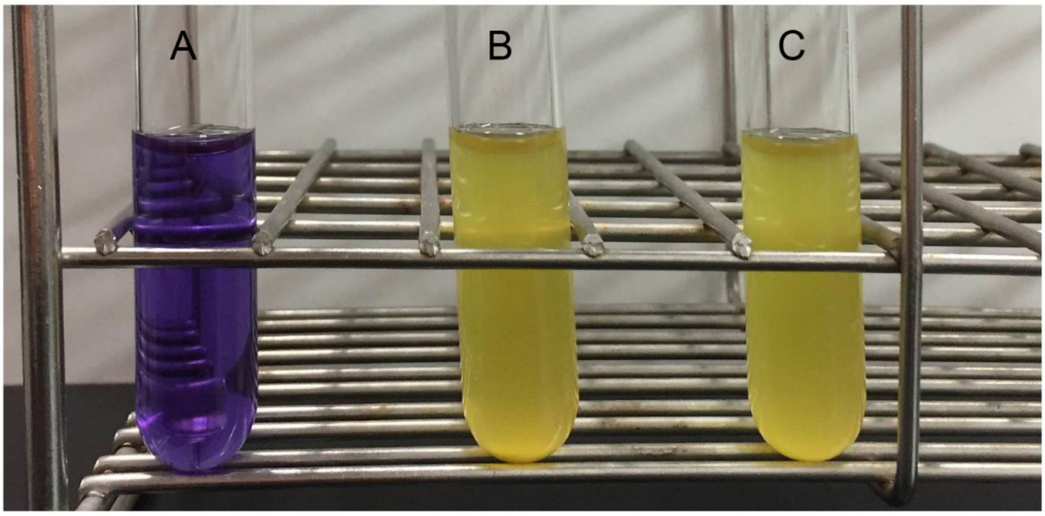

Figure S2. Color change of the pigmented modified Lim broth with bacterial growth. A loop-full of bacterial cells was used to inoculate 3 mL of the modified Lim broth. Uninoculated medium served as the negative control. The samples were incubated at 37°C for 24 h. (A) Broth without bacterial inoculation. (B) Broth inoculated with *S. agalactiae* strain KUGBS2rif. (C) Broth inoculated with *E. faecalis* strain KUEF08. After incubation, the culture broth color changed from violet to yellow in *S. agalactiae*- and *E. faecalis*-inoculated medium (B and C, respectively).

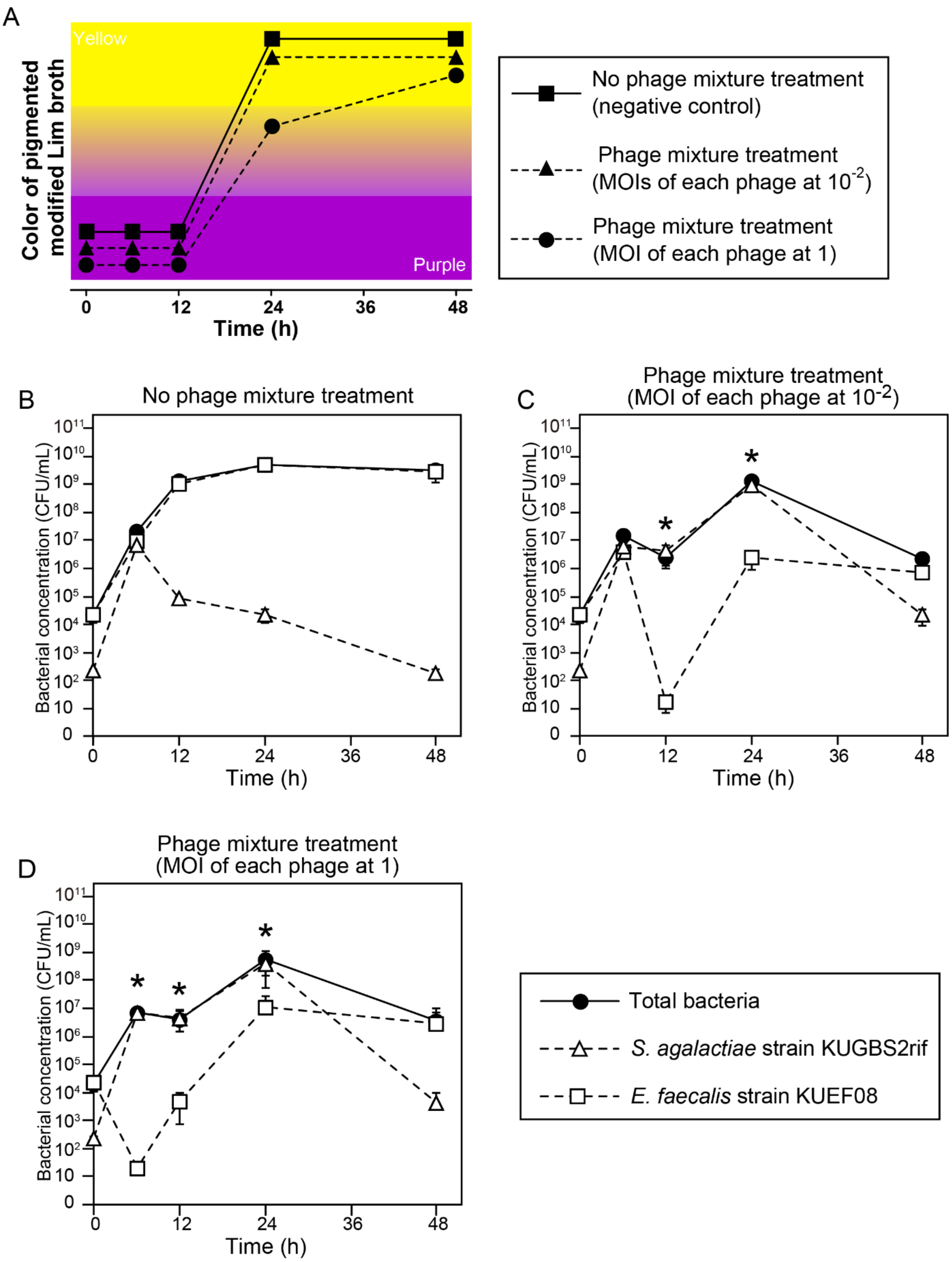

Figure S3. Effects of the phage mixture on bacteria inoculated in a commercially available *S. agalactiae* enrichment broth. *S. agalactiae* strain KUGBS2rif and *E. faecalis* strain KUEF08 were inoculated at ca. 3.0×10^2 CFU/mL and ca. 3.0×10^4 CFU/mL, respectively, in the

modified Lim broth. Two different dilutions of phage mixtures were added, with the density of each phage in the phage mixture of ca. 3.0×10^4 PFU/mL and ca. 3.0×10^2 PFU/mL. The MOIs of each phage to *E. faecalis* were 1 and 10^{-2}. As a negative control, THB was used instead of the phage mixture. The incubation was performed at 37°C. Changes in culture broth color and bacterial cell density (total bacteria, *E. faecalis*, and *S. agalactiae*) were recorded over time. (A) Color change of the enrichment broth. The experiments were repeated in triplicate. Supplementation with phage mixture at the highest density delayed the color change of enrichment broth. (B–D) Changes in bacterial cell density. Treatments: no phage treatment (B); or treatment with phages at 10^{-2} (C) and 1 (D) MOI of each phage to *E. faecalis.* Means with standard deviations (error bars) were calculated from triplicate experiments, and are plotted. Time points at which *S. agalactiae* density was significantly higher than *E. faecalis* density are indicated by an asterisk ($p < 0.01$; Student's *t*-test).

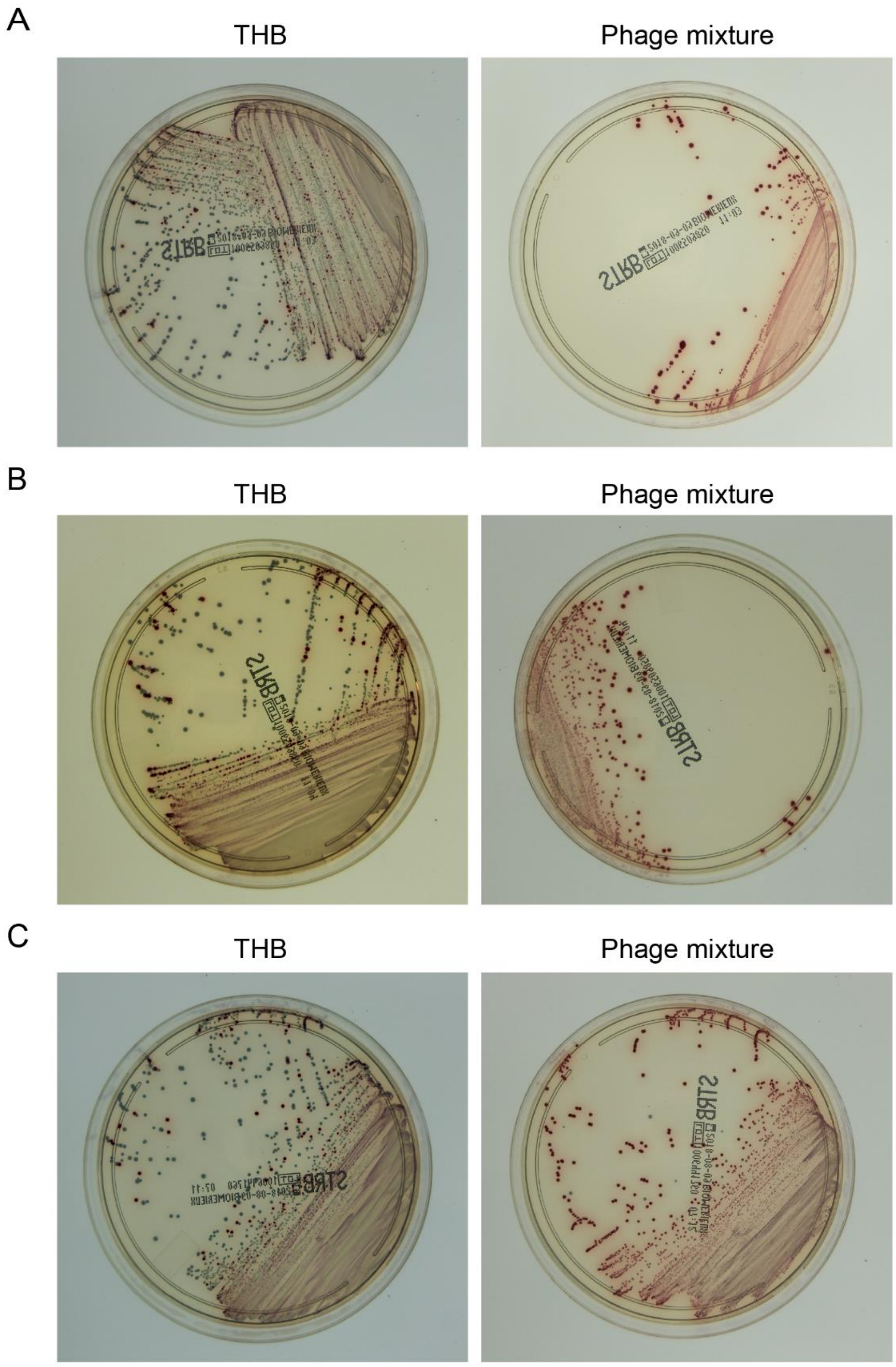

Figure S4. Bacterial identification on chromogenic agar after experimental enrichment co-culture of *S. agalactiae* and *E. faecalis*. Combinations of single strains of *S. agalactiae* and *E. faecalis* were inoculated in the modified Lim broth, and cultured together with THB or the phage mixture (MOI of each phage to *E. faecalis*: 10^{-1}). After enrichment culture, aliquots were plated on the chromogenic agar, and the resultant colonies evaluated. Colonies of *S. agalactiae*

and *E. faecalis* are red and blue, respectively. Left and right panels, photographs of chromogenic agar plates inoculated with aliquots of cultures treated with THB or phage mixture, respectively. Representative data for three out of five *S. agalactiae*–*E. faecalis* sets are shown, namely, KUGBS6–KUEF26 (A), KUGBS5–KUEF24 (B), and KUGBS3–KUEF03 (C).

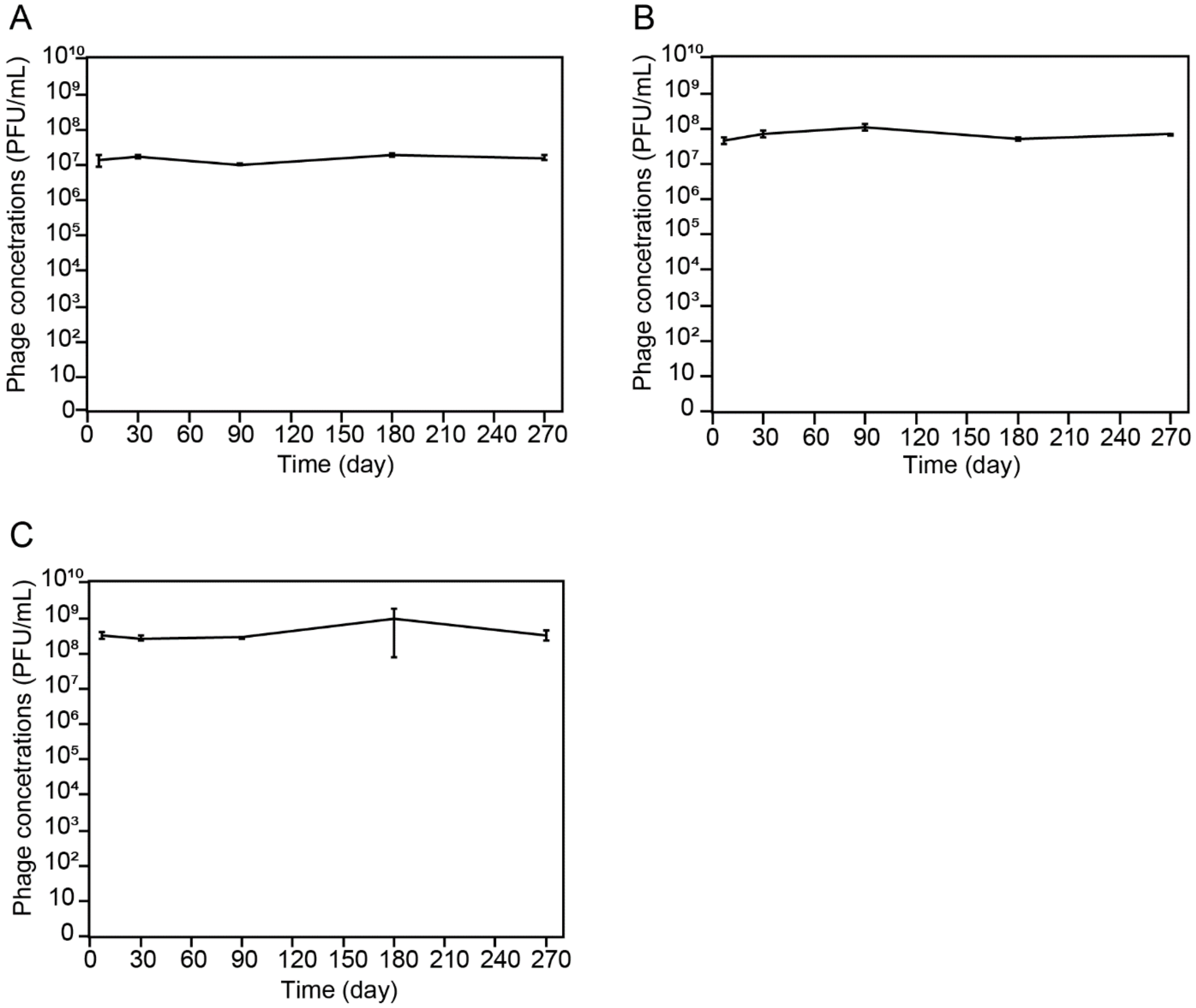

Figure S5. Phage stability. Phage suspensions were added to THB supplemented with 10.0 mg/l colistin sulfate, 10.0 mg/L metronidazole, and 15.0 mg/L nalidixic acid. The mixtures were stored at 4°C, and phage particle densities were determined by the double-agar plate method using an appropriate bacterial host. Changes in particle density of phiEF24C (A), phiEF17H (B), and phiM1EF22 (C) during incubation for 270 d at 4°C are shown. The densities were determined on days 7, 30, 90, 180, and 270. Means (straight lines) and standard deviations (error bars) were calculated from triplicate experiments.
